# Meta Analysis of Human AlzGene Database: Benefits and Limitations of Using *C. elegans* for the Study of Alzheimer's Disease and Co-morbid Conditions

**DOI:** 10.3389/fgene.2017.00055

**Published:** 2017-05-12

**Authors:** Behrad Vahdati Nia, Christine Kang, Michelle G. Tran, Deborah Lee, Shin Murakami

**Affiliations:** Department of Basic Sciences, College of Osteopathic Medicine, Touro University CaliforniaVallejo, CA, USA

**Keywords:** Alzheimer's disease (AD), amyotrophic lateral sclerosis (ALS), comorbidity, genome-wide association study (GWAS), meta-analysis, multiple sclerosis (MS), Parkinson's disease (PD), schizophrenia (SZ)

## Abstract

Human genome-wide association studies (GWAS) and linkage studies have identified 695 genes associated with Alzheimer's disease (AD), the vast majority of which are associated with late-onset AD. Although orthologs of these AD genes have been studied in several model species, orthologs in the nematode, *Caenorhabditis elegans*, remain incompletely identified, with orthologs to only 17 AD-related genes identified in the *C. elegans* database, WormBase. Therefore, we performed a comprehensive search for additional *C. elegans* orthologs of AD genes using well-established programs, including OrthoList, which utilizes four ontology prediction programs. We also validated 680 of the AD genes as a unique gene from the AlzGene database, including 431 genes (63%) that are predicted to have orthologs in *C. elegans*. Another 178 human AD genes (26%) were associated with one or more other neurological diseases, including amyotrophic lateral sclerosis, multiple sclerosis, Parkinson's disease, and schizophrenia. Of these, there were 105 genes (59%) with orthologs in *C. elegans*. Interestingly, three AD genes (*ACE, TNF*, and *MTHFR*) were associated with all four of the other neurological diseases. The human AD genes were enriched in three major ontology pathway groups, including lipoprotein metabolism, hemostasis, and extracellular matrix organizations, as well as in pathways that are amyloid related (NOTCH signaling) and associated with neural (neurotransmitter clearance) and immune (advanced glycation end-product receptor signaling and TRAF6-NF-kappaB) systems. Thus, the results from this study provide a potentially useful system for assessing comorbidities that may be associated with late-onset AD and other neurological conditions. The technical advantages and limitations of the ortholog searches are further discussed.

## Introduction

Alzheimer's disease (AD) is the most common cause of dementia but is currently without effective treatment options. Dementia is also caused by other neurological conditions, and increasing research and autopsy results reveal that many elderly patients exhibit dementia caused by more than one brain abnormality (Alzheimer's Association, 2015). We are interested in studying AD treatment in a more systematic, but personalized approach based on genomics. AD can be classified into early onset AD (EOAD) and late onset AD (LOAD). The human genetic makeup is one of the main susceptibilities for developing AD. Genome-wide association studies (GWAS) and linkage studies have identified 695 human genes associated with Alzheimer's disease (referred to as AD genes in this manuscript; www.alzgene.org; Bertram et al., 2007; Olgiati et al., 2011). AD genes are recognized as risk factors, but the causality is yet unknown.

EOAD affects patients before the age of 65 and is caused by mutations in APP, PSEN1, or PSEN2 (Campion et al., 1999). However, most cases (greater than 95%) of AD are LOAD patients who are over the age of 65 (Alzheimer's Association, 2015). Although the mechanism through which LOAD occurs remains unknown, there are certain genes that have been identified as risk factors, such as the presence of APOE-e4 increasing the chances of developing LOAD (Saunders et al., 1993). As such, it can be said that APOE-e4 and other genetic risk factors for LOAD, compounded with environmental and lifestyle choices, can influence the development of AD. Therefore, LOAD can be classified as a progressive multifactorial disorder.

Exactly how and to what degree the genes contribute to the development of AD is not well-understood. Direct studying of the human genes for AD is not an easy task and is very limited in practice. We reason that studying the genes in model systems, including the nematode *Caenorhabditis elegans*, is straightforward and ideal for an early stage study. *C. elegans*, the first multicellular organism to have its entire genome sequenced, possesses a genome with 20,317 protein coding genes (WormBase Release Letter WS252, 2011)^1^. From various estimates, roughly 42% of human genes seem to have orthologous counterparts in *C. elegans* (Palikaras and Tavernarakis, 2013), and between humans and *C. elegans*. The orthologs are from a common ancestral gene, and thus, are thought to have similar functions. The wormbase database list well conserved genes, for example the *daf-2* insulin/IGF-1 receptor gene (three human orthologs, INSR, INSRR, and IGF1R), the *age-1* phosphoinositide 3-kinase catalytic subunit gene (four human orthologs, PI3KA, PI3KB, PI3KC, and PI3KD).

This study aimed to validate the procedures that identify orthologs of human AD genes. As a first step, we identified *C. elegans* orthologs of human Alzgenes (i.e., genes listed in the AlzGene.org). Surprisingly, identifying C. *elegans* orthologs was not as straightforward as anticipated. It was in part due to variations among the ortholog gene pools generated by each search engine used. We utilized two criteria: a shared gene pool identified by all selected search engines (stringent pool), as well as a gene pool identified by each search engine (overall pool). We will use the numbers from the overall pool in this manuscript unless otherwise noted.

WormBase and OrthoList both identified overlapping and non-overlapping human gene pools. However, we were also surprised to find that OrthoList, which has consisted of a fixed number of human genes since 2013, identified more orthologs than the constantly updated WormBase. Interestingly, we found that the genes are not only associated with AD, but also presented as risk factor genes for other neurological diseases including Amyotrophic lateral sclerosis (ALS), Multiple sclerosis (MS), Parkinson's disease (PD), and Schizophrenia disease (SZ); three AD genes (*ACE, TNF*, and *MTHFR*) are associated with all four diseases, while 178 AD genes are associated with one or more of the diseases. This study provides cautions about the functional analysis in this model system as well as an importance of *C. elegans* gene counterparts that provides an insight into the mechanism underlying co-occurring human diseases, which may in part explain the complexity of AD diseases.

## Materials and methods

### Dataset

**Figure 1:**
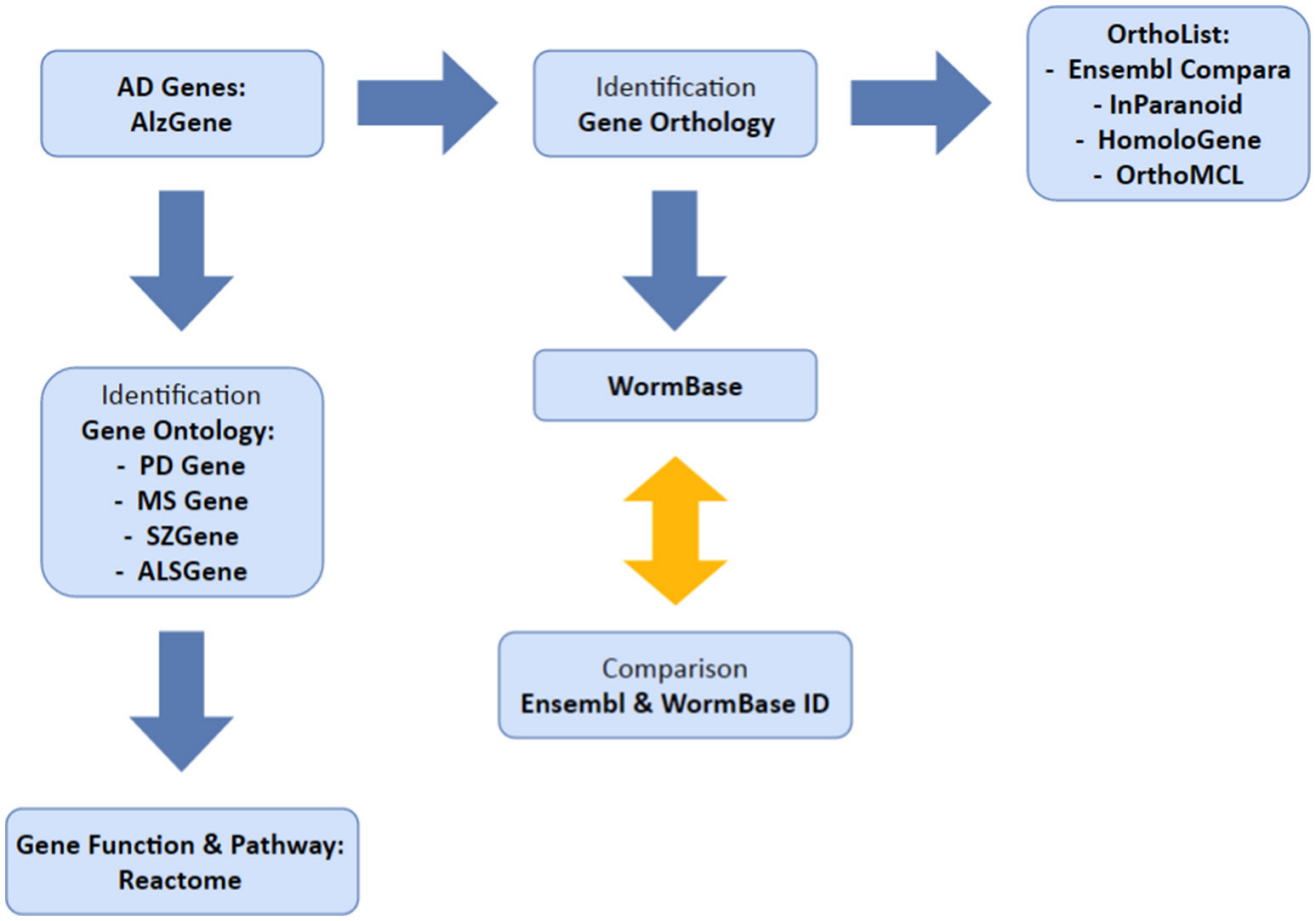
**Illustrates the methodology used in this study**. We selected our set of AD genes from AlzGene database and identified orthologs in *C. elegans* and genes common to multiple neurological disorders.

Our overall method has been summarized in Figure 1. We used the AlzGene database (www.alzgene.org; last accessed January 2017) to extract a list of identified human AD genes from GWAS and previous linkage studies. The AD genes were then used: (1) to identify *C. elegans* orthologs, using OrthoList and WormBase; (2) to correlate with the genes that are listed in four neurological disease databases (PDgene.org, MSgene.org, SZgene.org, and ALSgene.org). To avoid technical complications, Ensembl IDs were used for human genes and locus IDs for *C. elegans* genes. In the AlzGene database, each AD gene was linked to a number of positive or negative test results from each GWAS study, which were then used to validate the reliability of the data. Research outcomes listed as trends or inconclusive were not included in our study. The latest access to each search program below was on April 2016 unless otherwise noted.

### Orthology identification

#### WormBase

In order to find the appropriate ortholog counterparts of AD genes in *C. elegans*, we performed matching registry tests using WormBase (www.wormbase.org; accessed on 2016; Stein et al., 2001). WormBase is a consortium that contains biological and genomic information on *C. elegans* and other related nematodes. The database is regularly updated based on new research submissions and provides lists of homolog genes between *C. elegans* and other species. We experienced difficulty using the default search menu because it did not consistently provide the genes we were looking for. For this reason, we performed multiple searches using gene names, protein names, and locus IDs to generate a complete list. As an additional measure to avoid missing any genes, we also used BLASTP (protein-protein Basic Local Alignment Search Tool) match results from WormBase to confirm that the procured gene similarity default browser matched the registry results based on gene data (Wheeler et al., 2008).

#### OrthoList

In order to validate the completeness of WormBase's results, OrthoList was the second database we used to obtain a second list of orthologous AD gene counterparts in *C. elegans*. We also chose OrthoList as a control database because it contains a fixed number of genes (Aug. 2013) (Shaye and Greenwald, 2011). OrthoList is a database compiled from the meta-analysis of four unique programs which predict orthologous genes (Shaye and Greenwald, 2011; accessed on 2016). The four programs are: Ensembl Compara, InParanoid, Homologene, and OrthoMCL (described separately below). We then compared the matched registry test results produced from WormBase against those of OrthoList and identified the AD genes matched by both programs. The list of genes generated by OrthoList was also divided based on the number of *C. elegans* genes associated with each human AD gene. In cases where more than one *C. elegans* genes was associated with a single human gene, they would be labeled as orthologs with multiple WormBase IDs; if only a single *C. elegans* gene was associated with a single human gene, it would be labeled as an ortholog with a single WormBase ID.

#### Ensembl compara

It uses both sequence level and gene level analysis to obtain data on cross-species, and phylogenetic trees are used to represent such data. Their Protein trees include a protein associated with a specific gene based on NCBI BLAST+ e-values to assess the level of homology. These proteins are then clustered and aligned using different techniques (ensemblgenomes.org; accessed on 2016; Kersey et al., 2016).

#### InParanoid

It uses NCBI BLAST to calculate and create orthologous groups of two complete proteomes (Remm et al., 2001). Each orthologous group contains two seed orthologs that are determined by two-way best hits between the proteomes. Additional sequences are added to each group based on the closeness of the sequences in the proteomes to the corresponding seed orthologs (inparanoid51.sbc.su.se/cgi-bin/faq.cgi., 2001).

#### HomoloGene

It compares the protein and sequence makeup of different species with either a complete genome or at least 10,000 UniGene entries to create putative homology groups. BLASTP is used to assess the homology of the genes and different species are then divided based on their genomic makeup similarity (www.ncbi.nlm.nih.gov/homologene., 2007).

#### OrthoMCL

It uses BLASTP on proteins and computes the percent match of sequences based on their length (Li et al., 2003). A threshold is set for the BLAST results and only matches with e-values of less than 1e-5. Based on these results, possible ortholog, inparalog, and co-ortholog pairs are obtained. Lastly, OrthoMCL is used to cluster these pairs into groups (http://orthomcl.org/orthomcl/., 2014).

### Gene ontology and other neurological disease analyses

Ontology analysis was performed with PantherDB and UniProtKB, Swiss-Pro (www.ebi.ac.uk/QuickGO; Binns et al., 2009). The results of PantherDB were inconclusive due to either missing or incomplete lists of functions for individual genes. Thus, we used UniProtKB, Swiss-Pro in this study. The ontology for each gene was classified into three gene ontology (GO) sections: GO biological process, GO molecular function, and GO cellular component. The gene ontologies for the selected genes were categorized to determine possible common or shared ontologies.

Reactome analysis (www.reactome.org) is a type of ontology analysis combined with cluster analysis (Milacic et al., 2012; Fabregat et al., 2016). We used Cytoscape ver. 3.3.0 (Java version: 1.8.0_77) to run the Reactome software plugin, Reactome FIViz app5 (Wu et al., 2014). The version of the pathway database was Reactome v56 (released on March 26, 2016; last accessed on May 10, 2016). The Reactome FIViz was used to determine enrichment in the Functional Interaction (FI) network, the pathway enrichment of the genes of interest, followed by converting the results to interactomes. Statistics and false discovery rate (FDR) were calculated by the Reactome FIViz. Bonferroni correction was made to control familywise false positives, namely, type I error. The Reactome uses Ensembl Compara, which is one of the four ontology programs we used. Since four programs should provide a better overview of the orthologs, we did not use the ontology functions in Reactome FIViz. Twenty three general reactome pathway groups covered: cell cycle, cell-cell communication, cellular responses to stress, chromatin organization, circadian clock, developmental biology, DNA repair, DNA replication, extracellular matrix organization, gene expression, hemostasis, immune system, mitophagy, metabolism, metabolism of proteins, muscle contraction, neuronal system, organelle biogenesis, and maintenance, programmed cell death, reproduction, signal transduction, transmembrane transport of small molecules, and vesicle-mediated transport.

The list of AD Genes we compiled from the AlzGene database was also used to cross-reference the presence of any common genes with four other neurological diseases: Parkinson's disease (PD), multiple sclerosis (MS), schizophrenia disease (SZ), and amyotrophic lateral sclerosis (ALS). We adopted the following databases: PDGene (www.pdgene.org), MSGene (www.msgene.org), SZGene (www.szgene.org), and ALSGene (www.alsgene.org) to check for AlzGene counterparts in the aforementioned diseases. The selected genes were then organized based on their positive test and negative test results from their associated studies. For the ALSGene database, since the definite negative and positive results were not included in the database, we used the *p*-value from previous studies. If the *p*-value was < 0.05, we assumed the data was reliable and counted them as a positive result; studies with *p* > 0.05 were regarded as negative. It is important to note that the details regarding different genes were available for all of the neural disorders of interest, with an exception for ALS because the ALSGene database was separate from the main search engine.

## Results

In this study, we define homolog, ortholog, and paralog based on the definition that shows evolutionary relationships among genes (Fitch, 1970). A homolog, or a homologous gene, is related to another gene that comes from the same ancestral DNA. It includes orthologs and paralogs. Orthologs are genes generated by speciation. They are in different species and their functions may be retained during evolution. Paralogs are genes generated by a gene duplication event. They are in the same species. Orthologs are functionally conserved more likely than paralogs are (Li et al., 2005; Hulsen et al., 2006).

To identify orthologs, we performed a meta-analysis using two different databases embedded in WormBase and OrthoList. A keyword search of “Alzheimer” in the Wormbase database pulled out only 17 genes listed, the number of which may be underrepresented (Accessed January 2017). WormBase is a well-maintained consortium that includes constant updates and comprehensive information on *C. elegans* and related species (www.wormbase.org). OrthoList is a compendium of four separate orthology predicting programs that determine orthologous pairs between human and *C. elegans* genes (Shaye and Greenwald, 2011). OrthoList was chosen as a control database because it contains a fixed number of genes (Shaye and Greenwald, 2011). To further regulate and validate the overall experiments, we selected the AlzGene database to provide a set number of the AD genes (updated April 2011), allowing us to discern any variations among the different worm gene search programs we used. Due to the genome projects having been completed in human and *C. elegans*, we reasoned that the increases in gene numbers should be minimal.

### Comparison of predicted orthologs between wormbase and ortholist

**Figure 2:**
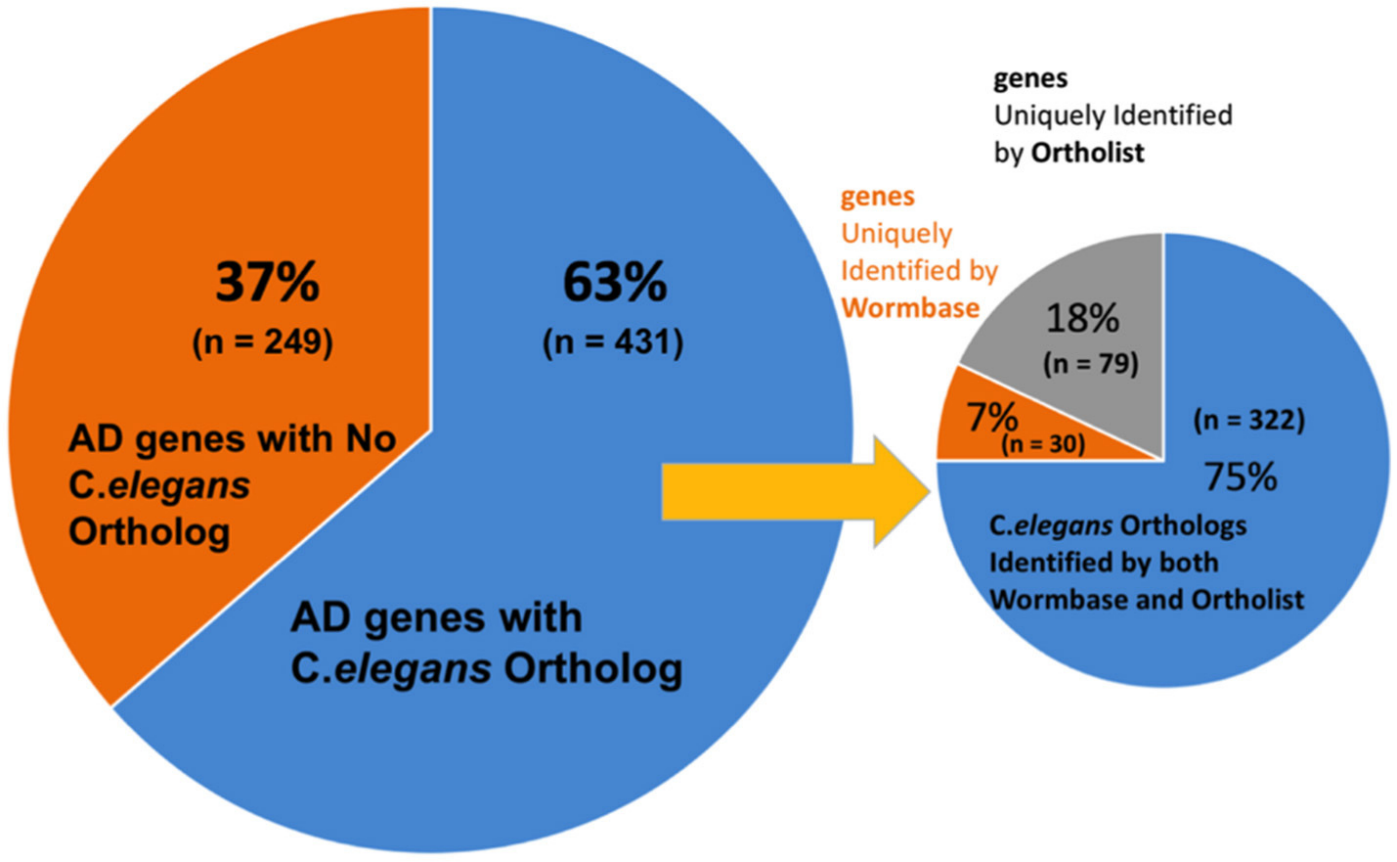
**Distribution ***C. elegans*** ortholog identification in WormBase and OrthoList**. Out of 680 AD genes, 431 were identified to have an orthologous match in *C. elegans*. A discrepancy was observed between the two databases: 30 orthologous matches were uniquely identified by WormBase and 79 from OrthoList.

We first validated the AlzGene database with 695 human genes identified by previous meta-analysis (Alzgene.org). We found that some genes in the AlzGene database were counted more than once, and omitting the redundancies resulted in a revised count of 680 human AD genes (Supplementary Table 1). These genes were then used to identify orthologs in *C. elegans* (Figure 2). A summary of predicted orthologs identified using WormBase and OrthoList is shown in Table 1 (also see Supplementary Table 2). Firstly, we used WormBase to match human genes between human and *C. elegans* as described in the method. We identified 431 out of 680 human AD genes (63%) as predicted orthologs (Figure 2). The number was relatively close to what was expected in *C. elegans* orthologs (42%; Palikaras and Tavernarakis, 2013).

**Table 1:** **Summary of the orthology searches using WormBase and OrthoList**.

**Database**	No. of Human AD genes that have *C. elegans* orthologs (%^*^)	**No. of C. *elegans* orthologs**	**No. of the human genes with positive results (%)**
WormBase	352 (52)	950	174
OrthoList	401 (59)	964	203
Specific to WormBase specific or to OrthoList	109 (16)	357	61
Common to WormBase and OrthoList (stringent pool^**^)	322 (47)	1088	158
Total (overall pool^***^)	431 (63)	1445	219

*Of 695 genes, 680 unique Alzgenes were used to identify C. elegans orthologous counterparts*.

* *The percentages out of 680 genes are shown*.

** *Stringent indicates stringent gene pool that is identified both by WormBase and by OrthoList*.

*** *Total indicates overall pool of the genes that are identified either by WormBase or by OrthoList. See also Supplementary Table 2*.

**Figure 3:**
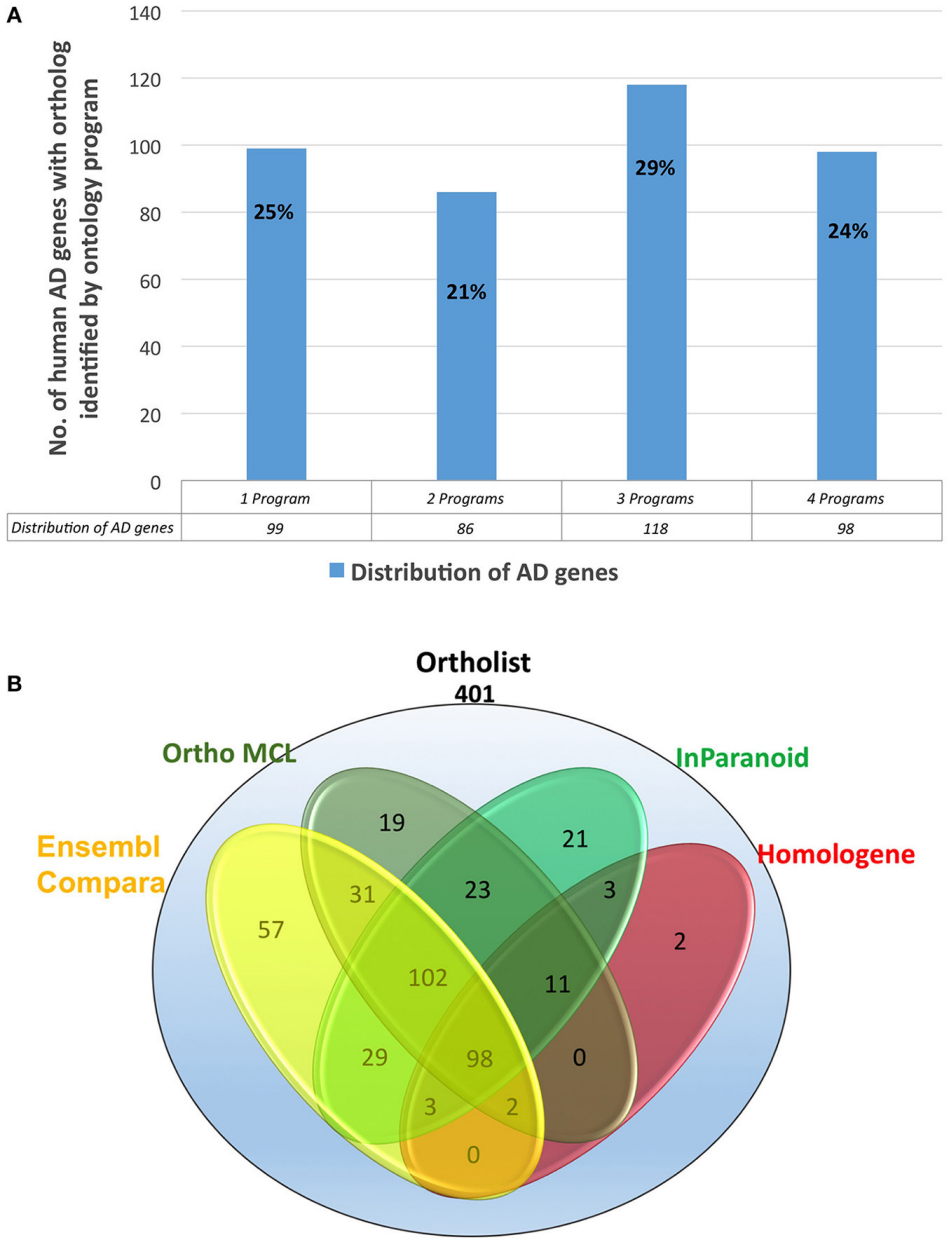
**(A)** Percentages of the distribution percentages of OrthoList's 401 AD gene match results among its four unique programs (Ensembl Compara, OrthoMCL, InParanoid, Homologene). **(B)** Numbers of the distribution of OrthoList's 401 AD gene match results.

Secondly, to further validate the results from WormBase, we expanded the search by using an additional search program, OrthoList (Shaye and Greenwald, 2011), which is a compendium of *C. elegans* genes that utilizes four orthology prediction programs (Ensembl Compara, InParanoid, Homologene, and OrthoMCL). Surprisingly, the OrthoList search identified more genes than the more updated WormBase. Of the unique 680 AD genes, OrthoList predicted 401 ortholog matches in *C. elegans* (59%; Figures 2, 3; Table 1), compared to 352 genes identified by WormBase (see above).

Thirdly, in order to again compare the results from WormBase with OrthoList, we associated each gene, using Ensembl ID. From the list of 352 ortholog matches produced from WormBase and 401 from OrthoList, both databases were in agreement for 322 of the AD genes predicted to have an orthologous match in *C. elegans* (Figure 1). And of the 322 common AD genes, 199 genes had a single WormBase ID (i.e., one Alzgene to one *C. elegans* gene), and 123 genes had multiple WormBase IDs (i.e., one Alzgene to multiple *C. elegans* genes). Our results showed that there is a relatively high variability (from 8.5 to 20%) between the two programs. Seventy-nine AD genes out of the total 401 ortholog matches (20%) were uniquely identified by OrthoList; 30 AD genes out of the total 352 ortholog matches (8.5%) were uniquely identified by WormBase (Table 1). Two hundred forty nine genes from the AlzGene database's list of 680 AD genes, did not have an ortholog in either of the databases. 19 out of the 249 genes did not have a corresponding Ensembl ID; this class was classified as non-orthologs.

### Comparison with ensembl compara, inparanoid, homologene, and OrthoMCL

We evaluated 401 orthologous gene match registry results generated by OrthoList by directly accessing the four orthology prediction programs used in it (i.e., Ensembl Compara, InParanoid, Homologene, and OrthoMCL). We then compared the data obtained from WormBase with the 4 programs. In order to ensure that genes selected by each search engine were orthologs, the Ensembl ID associated with the genes had to be identified by each search engine. Out of the 401 AD gene orthologs identified by OrthoList, each program separately contributed a total of 99 AD gene ortholog matches (25%), and 98 AD genes (24%) were identified by all four various programs used in the OrthoList database (Figures 2, 3; Table 1). Our observation of variability among four orthology programs was consistent with a previous study (Shaye and Greenwald, 2011).

### AD genes as risk factor for multiple neurological disorders

We also compared the AD gene list with the databases of four other neurological disorders, including ALS, MS, PD, and SZ. One hundred seventy eight AD genes out of 680 (26%) were found to be associated with one or more of the four neurological disorders of interest (Table 3; Supplementary Table 3); 105 out of 178 AD genes (59%), were human genes predicted to have an orthologous counterpart (Table 2; See Supplementary Table 1). We discovered that 3 out of the 105 AD genes were shared by all five neurological disorders: ACE, MTHR, and TNF. Furthermore, two out of the three genes, ACE and MTHFR, were human genes predicted to have *C. elegans* counterparts, *acn*-1 and *mthfr*-1. We further studied the GO of the genes shared in the five disorders (AD, ALS, MS, PD, and SZ), using UniProtKB, Swiss-Pro. However, we found numerous terms with general functions (ranging from 10 to 100 ontological terms) for each gene (data not shown). For the reason, we did not explore the ontological functions due to the general nature of the functions.

**Table 2:** **Summary of the human AD genes associated with one or more of four neurological disorders (PD, MS, SZ, and ALS)**.

**Gene**	**No. of the genes (%)**
AD genes associated with one or more of four neurological diseases	178/680^**^ (26)
AD genes above^*^ that have *C. elegans* ortholog(s)	105/178 (59)
AD genes above^*^ associated with all four diseases	3/178 (2)

* *Alzgenes with one or more of four neurological diseases*.

** *680 unique Alzgenes. See also Supplementary Table 3*.

**Table 3:** **Distribution of the 178 human AD genes that are associated with other disorders**.

**Neurological Disorder**	**No. of AD genes that are associated with the disorder (%)**	**No. of AD genes that are associated with the disorder and have *C. elegans* Orthologs (%)**
Amyotrophic lateral sclerosis	17/178 (10)	11/17 (65)
Multiple sclerosis	77/178 (43)	36/77 (47)
Parkinson's disease	80/178 (45)	45/80 (56)
Schizophrenia disease	87/178 (49)	51/87 (59)

### Reactome analysis of human AD genes

**Figure 4:**
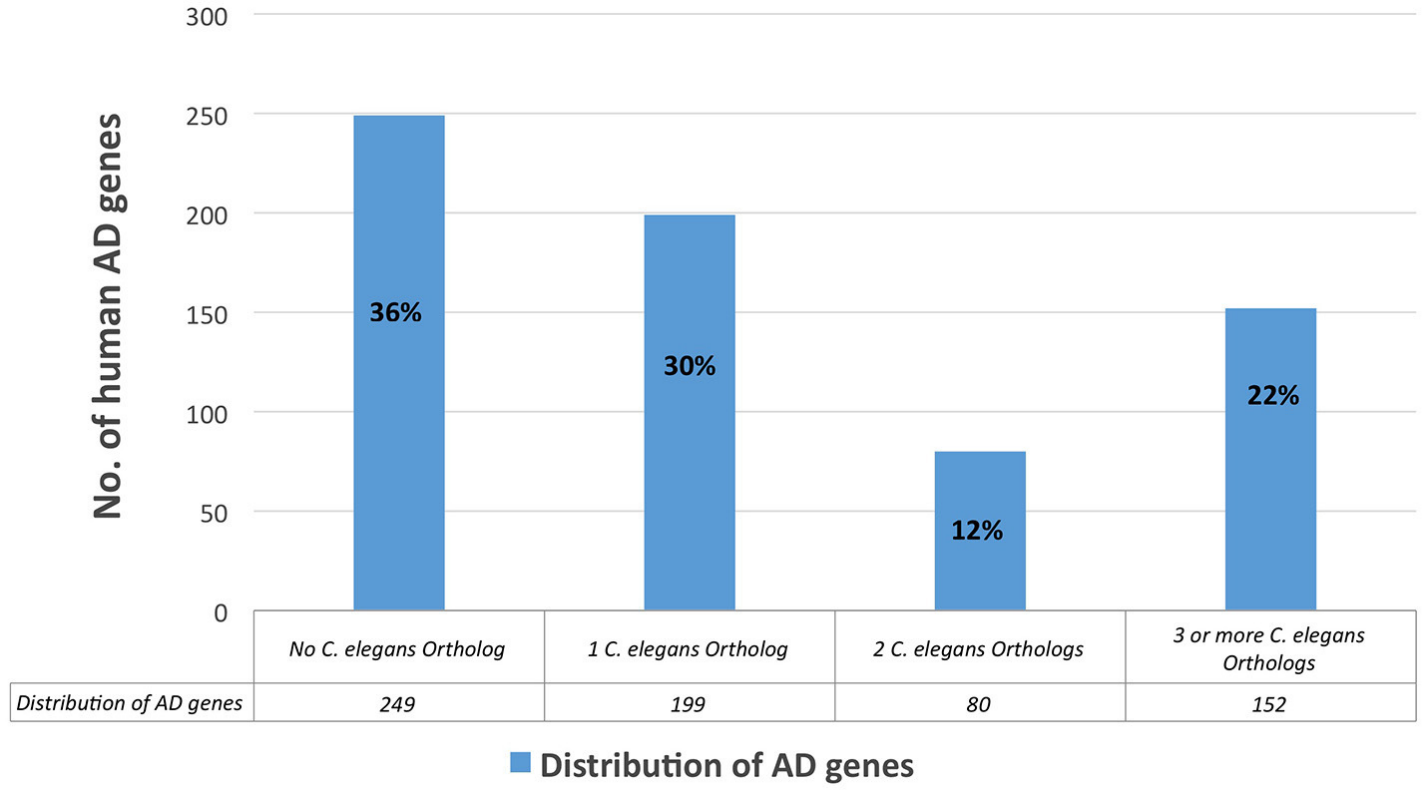
**Distribution of the all human AD genes, based on their corresponding number of *C*. *elegans* orthologs**.

We ran a Reactome analysis of the human AD genes. Reactome is a free and peer-reviewed pathway database that provides visualization and analysis of pathway knowledge to support basic research, genome analysis, and systems biology (Version, Reactome v56; released on March 26, 2016, and last accessed on April 22, 2016). Reactome database accommodates searches in 20 different species; in humans, it has 2,007 established pathways, covering 9,238 proteins. In order to concentrate our search most effectively, we organized the 680 human AD genes with positive test results from previous studies (positive genes) and ran the Reactome's pathway enrichment analysis for those genes only. Three hundred fifty six AD genes had positive results (positive genes) and 324 genes did not meet our criteria to be a positive gene (Figure 4). We then performed ontology analysis combined with cluster analysis, using Reactome knowledgebase (referred to as Reactome analysis; see Method). We set a threshold of the false discovery rate, FDR < 0.1, which is roughly the same as the probability being significant (*p* < 0.01). The results are summarized in **Table 5**.

Reactome analysis of the 356 positive AD genes suggested significant pathway enrichments of the AD genes in 3 general reactome groups (see Method for the list of all 23 general groups). They were metabolism, hemostasis, and extracellular matrix organization (*p* < 0.0015; FDR < 0.05; Bonferroni corrected *p* < 0.03). The control genes pulled out from the metabolism group were statistically significant, but we should consider the significance in metabolism to be dependent on the gene choice of GWAS studies. The control genes in hemostasis and extracellular matrix organization were not significant. We then further investigated specific reactome groups.

Our top result for the enriched pathway analysis was lipoprotein metabolism (FDR = 6.06E-10; Bonferroni corrected *p* = 1.78E-11), which was a sub-pathway topic under metabolism of lipids and lipoproteins, in addition to lipid digestion, mobilization, and transport (FDR = 1.00E-07; Bonferroni corrected *p* = 5.87E-09) in the hierarchy panel. Within lipoprotein metabolism, the analysis highlighted chylomicron-mediated lipid transport (FDR = 6.84E-07; Bonferroni corrected *p* = 8.03E-08) and HDL-mediated transport (FDR = 3.23E-06; Bonferroni corrected *p* = 5.70E-07). Lipoprotein metabolism involves 30 proteins, and within it, 14 were found to be shared in our positively tested AD genes (*p* = 7.73E-13; FDR = 6.06E-10; Bonferroni corrected *p* = 1.78E-11). Many of the hit-genes were found to overlap with a number of different pathways; for example, A2M was shared in lipoprotein metabolism, platelet degranulation, hemostasis, and degradation of the extracellular cellular matrix (**Table 5**).

We also further identified specific reactome groups within the general groups, including signal transduction, neural system, and immune systems (**Table 5**). In those general groups, specific groups were masked by other specific groups within. Therefore, we further investigated specific reactome groups.

**Figure 5:**
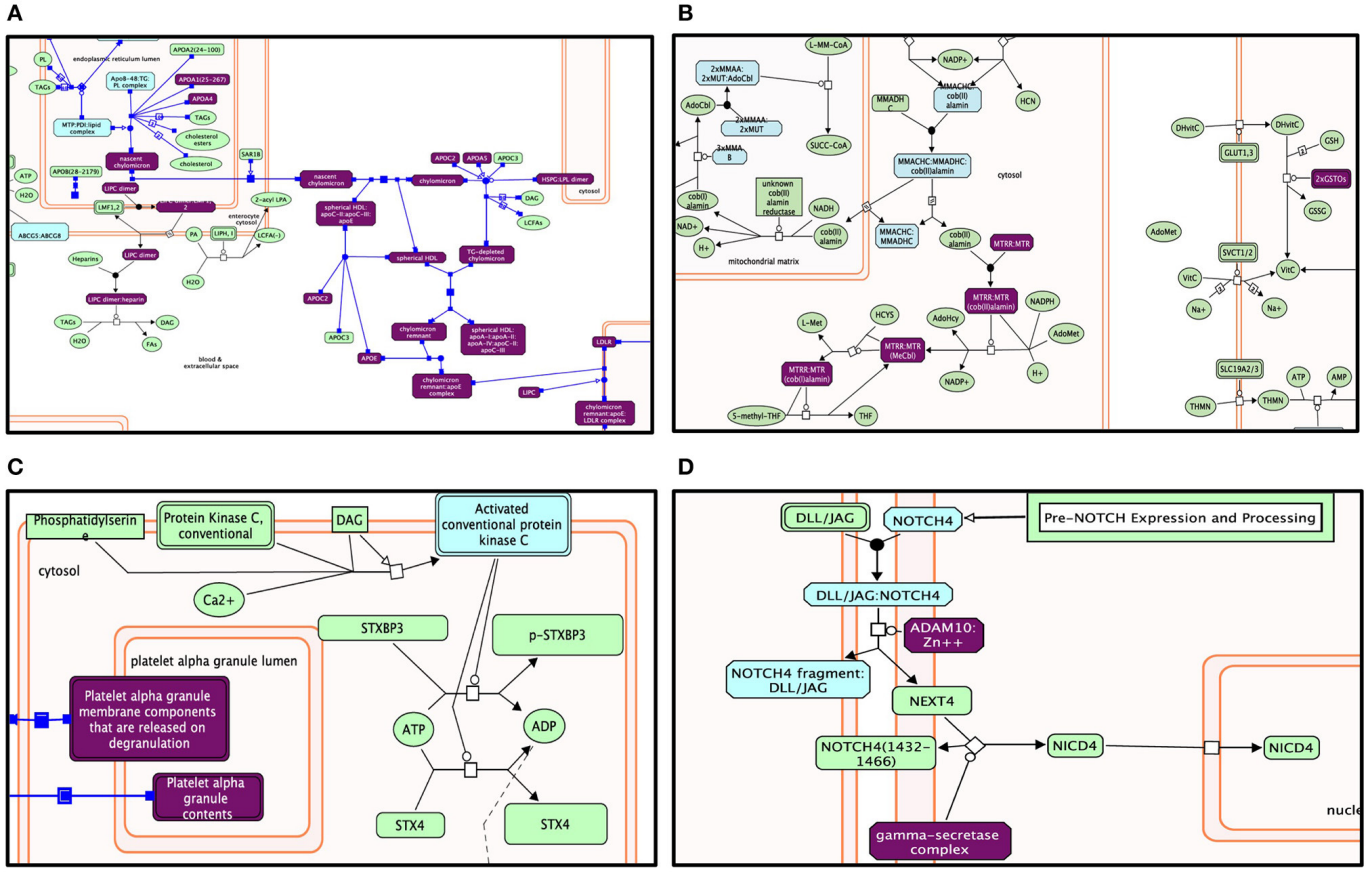
**Enriched pathway analysis: The entities in the diagram colored purple are hits within positive gene list**. Proteins are rectangles whereas elongated hexagons are complexes. Different Reactome pathways are highlighted in colors corresponding to their FDR values. **(A)** Lipid digestion, mobilization, and transport. **(B)** Metabolism of vitamins and cofactors. **(C)** Platelet activation, signaling, and aggregation. **(D)** Signaling by NOTCH4.

Surprisingly, in specific reactome groups, the amyloid-related group did not come up with the highest statistical significance. The group with the highest significance was metabolism related to lipoproteins, fat-soluble vitamins, and co-factors, as well as biological oxidations and angiotensins (*p* < 5.9E-03; FDR < 0.077; Bonferroni corrected *p* = 1.36E-01). There was no significance with metabolism related to carbohydrates (*p* = 0.72; FDR = 0.72; Bonferroni corrected *p* = 1.00), nucleotides (*p* = 0.85; FDR = 0.85; Bonferroni corrected *p* = 1.00), and amino acids (*p* = 0.44; FDR = 0.44; Bonferroni corrected *p* = 1.00) and lysin catabolism (*p* = 0.01; FDR = 0.10; Bonferroni corrected *p* = 0.23). The group with the second highest significance was hemostasis including platelet regulation (activation, signaling and aggregation) and fibrin clot, followed by amyloid-related groups having the third highest significance (NOTCH signaling, including activated NOTCH1 and signaling by NOTCH 2–4; *p* < 2.30E-04; FDR < 6.10E-03; Bonferroni corrected *p* = 5.29E-03). Other specific groups included but were not limited to amyloid-related (NOTCH signaling), neural systems (neurotransmitter clearance), and immune systems (advanced glycation end-product receptor signaling and TRAF6-NF-kappaB) (**Table 5**, Figure 5).

In addition to the NOTCH signaling, the general “Signal transduction” group demonstrated significance in visual phototransduction (related to retinoid metabolism and transport; *p* < 2.02E-09; FDR < 5.28E-07; Bonferroni corrected *p* = 4.65E-08). In contrast, some aging-related “groups” were not statistically significant, including signaling by insulin (*p* = 0.96; FDR = 0.96; Bonferroni corrected *p* = 1.00), regulation of IGF-1 (*p* = 0.093; FDR = 0.32; Bonferroni corrected *p* = 1.00), and post-NMDA activation events (including CREB) (*p* = 0.63; FDR = 0.63; Bonferroni corrected *p* = 1.00). The Parkinson disease-related group was also not significant (Pink-Parkin mediated mitophagy) (*p* = 0.48; FDR = 0.48; Bonferroni corrected *p* = 1.00). However, due to the nature of reactomes being incomplete, it is not clear whether or not negative results suggest a lack of interaction with AD. Distribution of the control AD genes among reactome groups was not similar, ruling out the possibility that the top three reactome groups were selected by chance.

**Figure 6:**
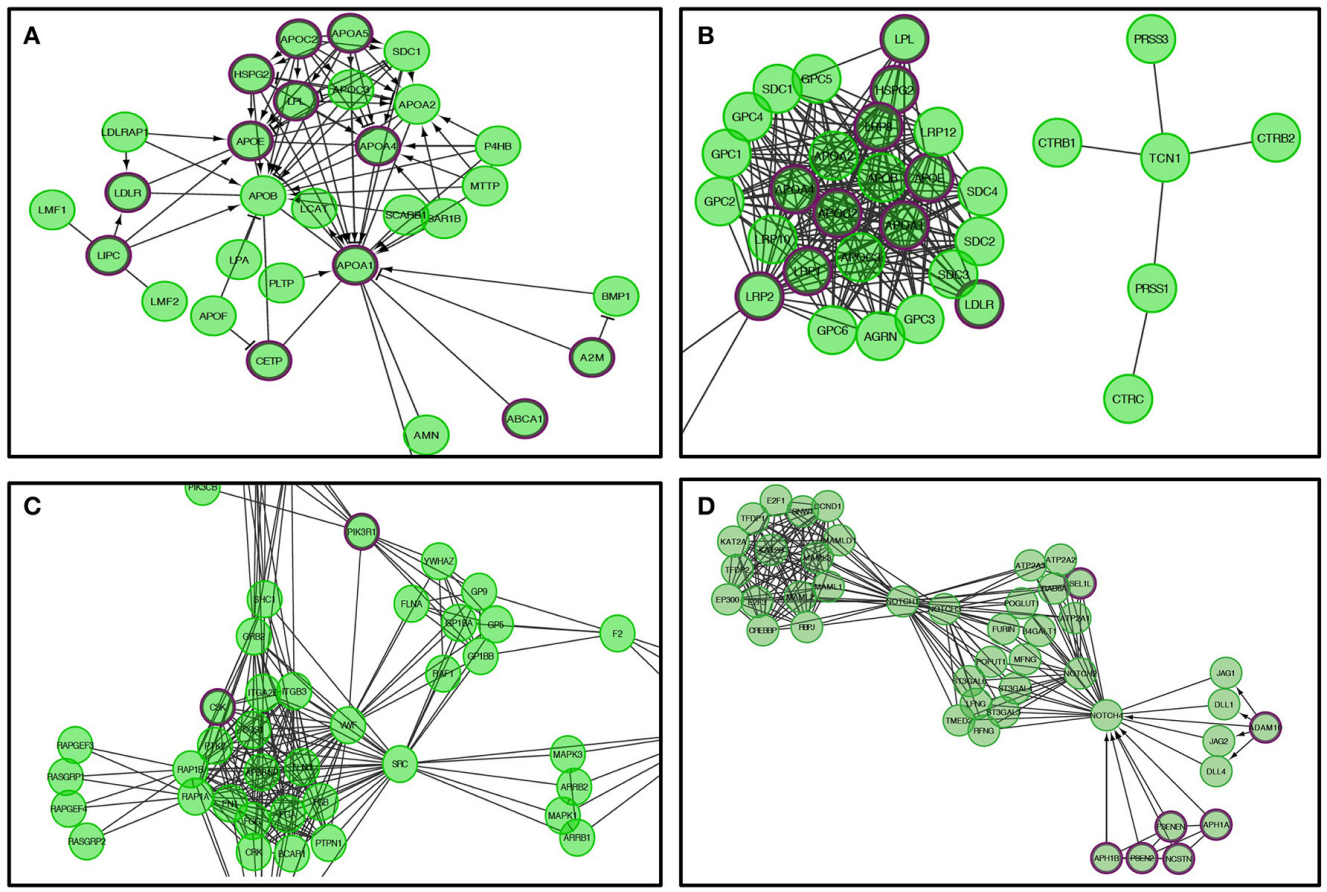
**The Reactome pathways displayed in Figure 5 were converted into a functional interaction network using FI network view**. Sub-pathways within the original pathway diagrams were extracted into the FI network as well. Hit genes are displayed in a thick purple border in the FI network view for a hit pathway. **(A)** Lipid digestions, mobilization, and transport. **(B)** Metabolism of vitamins and cofactors. **(C)** Platelet activation, signaling, and aggregation. **(D)** Signaling by NOTCH4.

Genes within our positively tested AD genes for lipid digestion, mobilization, and transport (Figures 5A, 6A) included the following sub-processes: arachidonic acid metabolism, fatty acid metabolism, triacylglycerol metabolism, ketone body metabolism, cholesterol biosynthesis, regulation of biosynthesis by SREBP (SREBF), bile acid and bile salt metabolism, metabolism of steroid hormones, sphingolipid metabolism, and phospholipid metabolism. Under metabolism of vitamins and cofactors, hit genes included metabolism of water and fat-soluble vitamins, as well as cofactors (Figures 5B, 6B). Hit genes involved in platelet activation, signaling, and aggregation were involved in GP1b-IX-V activation signaling, effects of PIP2 hydrolysis, platelet aggregation (plug formation), thrombin signaling via proteinase-activated receptors (PARs), GPVI-mediated activation cascade, and responses to elevated platelet cytosolic Ca^2+^ (Figures 5C, 6C). Pathway enrichment results for NOTCH signaling showed positive AD genes associated with pre-NOTCH expression and processing, in addition to signaling processes by NOTCH1, 2, 3, and 4. We elected to show signaling by NOTCH4 in particular because of its FDR value of 8.240E-5 (Bonferroni corrected *p* = 2.92E-05) (Figures 5D, 6D).

## Discussion

In this study, we identified 431 human AD genes (overall pool) and 322 genes (stringent pool) that have *C. elegans* counterparts. Given that only 17 genes are listed as Alzheimer's disease related genes in Wormbase (accessed January 2017), our results dramatically increase the number of the genes in the criterion.

We have performed a meta-analysis of the fixed number of human AD genes to validate fluctuations among current programs available. For this reason, we used the AlzGene database. We used two different programs to identify *C. elegans* orthologs: WormBase and OrthoList. WormBase has been constantly updated, while OrthoList has fixed gene datasets based on the assumption that there are minimal updates in the number of genes for *C. elegans*. However, despite our prediction that WormBase would provide a comprehensive list of orthologs, OrthoList contributed more genes than WormBase. Moreover, there was a relatively high amount of variability between WormBase and OrthoList, totaling in 16% (Shaye and Greenwald, 2011).

In addition, we discovered that a relatively high percentage of human AD genes had orthologous counterparts in *C. elegans* (59%). This percentage is consistent with a previous study: 42% of human genes have orthologs in *C. elegans*, and approximately 65% of human genes that cause disease have a counterpart in *C. elegans* (Palikaras and Tavernarakis, 2013). Our findings will also make a major contribution to WormBase's section of “Disease Ontology, Alzheimer's disease,” where there are currently 10 genes in the section of “Genes used as experimental models” and 8 genes in the section of “Genes identified as potential models” (WormBase accessed in April 2016). Our results from this study will add a new gene set of more than 400 genes.

Each particular search engine from OrthoList picked up a different number of predicted orthologs between the human and *C. elegans* genomes. This was not surprising because each search engine utilizes different parameters and methods to collect worm data, followed by a distinct set of criteria to identify predicted orthologs (described in the Method). Homologene had the least number of genes identified as orthologs compared to the three other search engines. Although the reason remains unclear, we speculate that the lower number may be due to higher specificity for the values set as a selection parameter for orthologs; the procedure is essentially a BLAST search looking at the genome sequences across different species. Ensembl Compara provided the most comprehensive orthologous search by creating a list of 322 registry matches out of 401 (Table 4).

**Table 4:** **Distribution of all 401 AD genes by OrthoList's four orthology search programs**.

	**Ensembl Compara**	**InParanoid**	**Homologene**	**OrthoMCL**
Total identified by each search program	321	290	119	286
Unique to each search program	56	21	2	19

More importantly, 178 of the AD genes (26% of total AD genes) were associated with one or more of four different neurological disorders (PD, MS, SZ, and ALS). Thus, approximately a quarter of the AD genes are associated with multiple diseases, which may account for some aspects of the complexity observed in patients with AD and other neurological disorders. Additionally, 3 AD genes were identified in all four diseases, of which 2 have orthologs in *C. elegans*. This raises the possibility that different neurological diseases may develop through similar mechanisms and the genes shared between them can be the common link. A closer study of the three genes: *ACE, MTHR*, and *TNF* functions may reveal the overlapping pathways that can lead to the development of such diseases. Tumor necrosis factor (*TNF*) is associated with insulin resistance and inflammation, which are characteristics found in Type II diabetes and can be a contributing factor that can lead to the pathogenesis of AD (de la Monte and Wands, 2008). A previous study published in 2011 focused on genes that were well-associated with LOAD from the AlzGene Database and analyzed the shared pathways that included *TNF* in their list of top 15 genes. *TNFalpha* is a gene involved in immune function and implicated in neuroinflammation, which is a downstream effect of beta-amyloid deposits that leads to the activation of microglia and potentially results in increasing tissue damage (Wang et al., 2015).

Although our meta-analytic data for the positive AD genes demonstrated involvement in a broad range of biological pathways, concurrent with previous studies, some of our most significant GO analysis were strongly associated with lipoprotein metabolism, lipid digestion, mobilization, and transport; these overlapped in every gene except for one, *CAV1* (Table 5). This adds to the consistent support of cholesterol's connection and importance in the pathogenesis of AD given that the brain has the highest content of cholesterol (~20%) in ratio to total body cholesterol (Puglielli et al., 2003).

**Table 5:** **Reactome pathway enrichment analysis results**.

**Pathway**	**No. of total proteins in pathway**	**No. of hits in pathway**	***p*-value**	**FDR value**	**Hit gene**
Lipoprotein metabolism	30	14	7.73E-13	6.06E-10	*A2M, APOE, ABCG1, LDLR, CETP, HSPG2, APOA4, APOA1, APOA5, APOC2, ABCA1, ALB, LPL, LIPC*
Lipid digestion, mobilization, and transport	56	15	2.55E-10	1.00E-07	*A2M, APOE, ABCG1, LDLR, CETP, HSPG2, APOA4, APOA1, APOA5, CAV1, APOC2, ABCA1, ALB, LPL, LIPC*
Retinoid metabolism and transport	37	12	2.02E-09	5.28E-07	*APOE, LDLR, HSPG2, LRP1, LRP8, LRP2, APOA4, TTR, APOA1, LRAT, APOC2, LPL*
Chylomicron-mediated lipid transport	17	9	3.49E-09	6.84E-07	*APOE, LDLR, HSPG2, APOA4, APOA1, APOA5, APOC2, LPL, LIPC*
Metabolism of fat-soluble vitamins	42	12	8.13E-09	1.28E-06	*APOE, LDLR, HSPG2, LRP1, LRP8, LRP2, APOA4, TTR, APOA1, LRAT, APOC2, LPL*
HDL-mediated lipid transport	15	8	2.48E-08	3.23E-06	*A2M, APOE, ABCG1, CETP, APOA1, APOC2, ABCA1, ALB*
Metabolism of vitamins and cofactors	114	17	8.87E-08	9.94E-06	*PTGS2, APOE, LDLR, HSPG2, LRP1, LRP8, LRP2, MTHFD1L, APOA4, TTR, APOA1, LRAT, APOC2, LPL, MTR, MTHFR, GSTO2*
Platelet degranulation	78	14	1.42E-07	1.39E-05	*A2M, APP, SERPINE1, CD36, SERPINF2, SERPINA1, TF, IGF1, F13A1, TGFB1, APOA1, CLU, ALB, HSPA5*
Response to elevated platelet cytosolic Ca2+	83	14	2.97E-07	2.59E-05	*A2M, APP, SERPINE1, CD36, SERPINF2, SERPINA1, TF, IGF1, F13A1, TGFB1, APOA1, CLU, ALB, HSPA5*
Platelet activation, signaling and aggregation	203	21	1.08E-06	8.24E-05	*A2M, APP, GAB2, SERPINE1, CD36, SERPINF2, FCER1G, SERPINA1, TF, DGKB, IGF1, GNA11, F13A1, TGFB1, APOA1, CLU, ALB, CSK, LCK, HSPA5, PIK3R1*
Signaling by NOTCH4	11	6	1.27E-06	8.24E-05	*PSENEN, ADAM10, PSEN2, APH1A, APH1B, NCSTN*
Signaling by NOTCH3	11	6	1.27E-06	8.24E-05	*PSENEN, ADAM10, PSEN2, APH1A, APH1B, NCSTN*
Metabolism	1551	77	1.37E-06	8.24E-05	*A2M, PTGS2, APOE, SGPL1, RXRA, GSTT1, CD36, DLD, HSD11B1, MAOA, ABCG1, HMGCS2, ARSB, PPARA, LDLR, PPARG, NOS3, CETP, MT-ATP8, DHCR24, COX15, AGPAT1, GNB3, ALDH18A1, DPYS, AGT, MT-CYB, MT-ATP6, NAT2, HSPG2, PCK1, GAPDHS, LRP1, LRP8, LRP2, DNM2, HMGCR, MTHFD1L, GSTM1, APOA4, TTR, GSTM3, APOA1, APOA5, NQO1, LRAT, CAV1, APOC2, ABCA1, ALB, CYP19A1, SREBF1, LPL, FDPS, MTR, ALDH2, HMOX1, ACAD8, MT-ND4, MT-ND6, MT-CO2, MT-CO3, MT-ND1, GSTP1, MT-ND2, MT-ND3, COMT, MTHFR, ACAN, GSTO2, GSTO1, GAPDH, PIK3R1, DLST, ALOX5, LIPC, CBS*
Hemostasis	454	32	6.50E-06	3.64E-04	*A2M, APP, GAB2, SERPINE1, CD36, SERPINF2, FCER1G, PLAT, PLAU, SERPINA1, NOS3, TP53, TF, DGKB, NOS1, IGF1, LRP8, GNA11, F13A1, MMP1, TGFB1, APOA1, CAV1, CLU, ALB, CSK, CDK5, LCK, HBG2, HSPA5, PIK3R1, OLR1*

*ACE* is involved in the Renin-Angiotensin-Aldosterone system and implicated in microvascular complications in Diabetes III (MVCD3) and ischemic strokes. MVCD3 include pathological conditions that result as a consequence of diabetes mellitus and include diabetic retinopathy, end-stage renal disease, and diabetic neuropathy. Low CSF ACE protein levels have been correlated with increased alpha-beta accumulation in the brain (Jochemsen et al., 2014).

Previously we have proposed to include feedback from patients into research when studying health (Murakami and Halperin, 2014) and provided an example (Murakami, 2016). Similarly, this study was performed in discussion with Alzheimer's patients. We incorporated the critique that there might be a more meaningful approach to AD patients than simply finding a pool of the AD genes. The discussion prompted us to explore co-morbidities that might be associated with AD. We found 178 human genes associated with one or more of neurological disorders. Identifying common links between the different neurological diseases may be the key to developing effective treatments for multiple diseases simultaneously. We hope to stimulate basic science research with more focus on patients.

## Author contributions

All authors listed, have made substantial, direct and intellectual contribution to the work, and approved it for publication.

## Funding

The work was supported by the research funds from Master of Science in Medical Heath Sciences, Touro University California (TUC) and by the work-study funds from TUC.

### Conflict of interest statement

The authors declare that the research was conducted in the absence of any commercial or financial relationships that could be construed as a potential conflict of interest.

## Footnotes

^1^WormBase Release LetterVer WS227 (2011). 10 August Retrieved 2013-11-19.

## Abbreviations

AD: Alzheimer's disease
ALS: Amyotrophic lateral sclerosis
GWAS: Genome-wide association study
MS: Multiple Sclerosis
PD: Parkinson's disease
SZ: Schizophrenia disease.
